# Seroprevalence of SARS-CoV-2 among Blood Donors and Changes after Introduction of Public Health and Social Measures, London, UK

**DOI:** 10.3201/eid2707.203167

**Published:** 2021-07

**Authors:** Gayatri Amirthalingam, Heather Whitaker, Tim Brooks, Kevin Brown, Katja Hoschler, Ezra Linley, Ray Borrow, Colin Brown, Nick Watkins, David J. Roberts, Danielle Solomon, Charlotte M. Gower, Olivier le Polain de Waroux, Nick J. Andrews, Mary E. Ramsay

**Affiliations:** Public Heath England, London, UK (G. Amirthalingam, H. Whitaker, K. Brown, K. Hoschler, C. Brown, D. Solomon, C.M. Gower, O. le Polain de Waroux, N.J. Andrews, M.E. Ramsay);; Public Health England, Porton Down, UK (T. Brooks);; Public Health England, Manchester, UK (R. Borrow);; National Health Service Blood and Transplant, Cambridge, UK (N. Watkins);; National Health Service Blood and Transplant, Oxford, UK (D.J. Roberts);; Radcliffe Department of Medicine, University of Oxford, Oxford (D.J. Roberts)

**Keywords:** COVID-19, coronavirus disease, SARS-CoV-2, severe acute respiratory syndrome coronavirus 2, viruses, respiratory infections, zoonoses, serology, surveillance, seroprevalence, England, United Kingdom, antibody

## Abstract

We describe results of testing blood donors in London, UK, for severe acute respiratory disease coronavirus 2 (SARS-CoV-2) IgG before and after lockdown measures. Anonymized samples from donors 17–69 years of age were tested using 3 assays: Euroimmun IgG, Abbott IgG, and an immunoglobulin receptor-binding domain assay developed by Public Health England. Seroprevalence increased from 3.0% prelockdown (week 13, beginning March 23, 2020) to 10.4% during lockdown (weeks 15–16) and 12.3% postlockdown (week 18) by the Abbott assay. Estimates were 2.9% prelockdown, 9.9% during lockdown, and 13.0% postlockdown by the Euroimmun assay and 3.5% prelockdown, 11.8% during lockdown, and 14.1% postlockdown by the receptor-binding domain assay. By early May 2020, nearly 1 in 7 donors had evidence of past SARS-CoV-2 infection. Combining results from the Abbott and Euroimmun assays increased seroprevalence by 1.6%, 2.3%, and 0.6% at the 3 timepoints compared with Euroimmun alone, demonstrating the value of using multiple assays.

The first confirmed cases of coronavirus disease (COVID-19) in the United Kingdom were identified at the end of January 2020. As cases increased across all regions, surveillance data indicated that the epidemic was progressing more rapidly in London than the rest of the United Kingdom. In response to the increase in cases, hospitalizations, and deaths, the United Kingdom introduced a series of measures to limit transmission, beginning March 12, 2020 (week 11); persons with a continuous cough or fever were advised to self-isolate for 7 days, school trips abroad were cancelled, and at-risk groups were advised to avoid cruises. These measures culminated in the implementation of legally enforceable public health and social measures (i.e., lockdown) beginning March 23 (week 13) (*1*).

Despite the reporting of a range of surveillance data in England, including laboratory-confirmed cases, primary-care consultations, hospital and intensive care unit (ICU) admissions, and deaths (*2*), much remains unknown about the magnitude of infection with severe acute respiratory syndrome coronavirus 2 (SARS-CoV-2) virus in the population, the key drivers of transmission, and the incidence of asymptomatic or mildly symptomatic infection within the UK population thus far.

Serologic estimates are critical to better understand epidemiologic trends and help inform policy options to control disease. These estimates also provide a denominator for estimating severity measures, such as infection fatality and infection hospitalization ratios, and to help clarify the epidemiology of COVID-19 in the population.

Early in the pandemic data from population-based seroepidemiologic studies were limited (N. Bobrovitz et al., unpub. data, https://doi.org/10.1101/2020.05.10.20097451), and how the prevalence of SARS-CoV-2 infection varies by age was not well understood. Much remains unknown about the dynamics of the antibody response to SARS-CoV-2. The existing serologic assays target different viral proteins, and IgG responses to these proteins are likely to appear at different stages of the immune response, potentially resulting in some assays preferentially identifying those persons seroconverting earlier or later in the course of an infection (*3*); these differences are an important factor when interpreting data from seroprevalence studies. In this article, we describe the results of testing whole blood donors in London, UK, who were anonymously tested as part of the national public health response to COVID-19. These tests were conducted using 3 different serologic assays at 3 timepoints during the epidemic that reflect transmission prelockdown, perilockdown, and immediately post lockdown.

## Methods

### Data Collection

#### Sample Selection

A program of collecting plasma samples each week through the National Health Service Blood and Transplant Services from healthy 17–69 year old persons donating whole blood was initiated on March 23, 2020, at epidemiologic week 13. The minimum interval between serial donations was 12 weeks for men and 16 weeks for women. An average of 10,683 whole blood donations were received per month in London during March–July 2020.

Given the evidence of the scale of the epidemic in London, enhanced testing of London donors was implemented with donor samples from London collected during week 13 (period 1, beginning March 23), weeks 15–16 (period 2), and week 18 (period 3). Approximately 1,000 fully anonymized donations were obtained for each collection. The demographic information available from each donor included age, sex, and area of residence.

Blood donors are healthy persons who are excluded from donating if they experienced any acute illness for >2 weeks before donating blood. In addition, specific donor exclusion criteria for coronavirus have been introduced (14 days postinfection at the time of the study, which was extended to 28 days starting June 8) (*4*). Given the standard symptomatic period, the exclusion criteria described and the fact that antibodies might take >2 weeks to be detectable, prevalence estimates among blood donors probably reflect infection prevalence >2–4 weeks before samples were taken.

To undertake the validation of test sensitivity, samples from recovering case-patients are required. To obtain convalescent serum samples from case-patients in the community, Public Health England (PHE) initiated an active request for samples from persons with PCR-confirmed cases reported early in the epidemic. These persons were asked to attend their general practitioner approximately 3–5 weeks after illness onset to provide a convalescent serum sample (N.L. Boddington et al., unpub. data, https://doi.org/10.1101/2020.05.18.20086157). Crucially, these cases were detected in the containment phase, when testing was based on epidemiologic factors such as travel. These cases should therefore be a better reflection of mild and asymptomatic infections that would not otherwise be picked up by routine testing, which was based predominantly on testing hospitalized patients at the 3 timepoints.

To evaluate specificity, serum samples collected before the circulation of SARS-CoV-2 also were tested. This testing was done on residual serum samples taken in 2018 and provided by the Sero-Epidemiology Unit (SEU) at PHE, Manchester (*5*), and the Royal College of General Practitioners Research and Surveillance Centre (*6*). All samples were processed and stored at the SEU.

#### Serologic Assays

We tested samples on 2 commercial assays according to the manufacturers’ instructions. Initial testing was conducted by using the SARS-CoV-2 ELISA IgG assay from Euroimmun (https://www.euroimmun.com) targeting the S1 domain, including the receptor-binding domain (RBD); testing was conducted by using the SARS-CoV-2 IgG for use on the Architect platform (Abbott, https://www.molecular.abbott) targeting the nucleoprotein. Samples were tested individually and reported according to the manufacturers’ criteria. We defined Euroimmun results of 0.8 to <1.1 as equivocal and >1.1 as reactive. We defined Abbott results of >1.4 as reactive; we also defined an equivocal range of 0.8 to <1.4 for presentation of validation data.

The third assay was an in-house assay developed in the Virus Reference Department at PHE, also used retrospectively. For this ELISA, we purchased the commercial recombinant RBD subunit of the SARS-CoV-2 spike protein from SinoBiological Inc. (https://www.sinobiological.com), which we expressed in HEK293 cell culture with a C-terminal mouse Fc tag (Arg319-Phe541(V367F) (GenBank accession no. YP_009724390.1). We coated Nunc MaxiSorp (Nunc, https://www.thermofisher.com) flat-bottomed, polystyrene, 96-well microtiter plates by diluting 20 ng recombinant protein per well in sterile phosphate-buffered saline; pH 7.2 + SD 0.05 (Gibco, https://www.thermofisher.com) at 4°C–8°C for a minimum of 16 hours. We diluted serum at a final dilution factor of 1 in 100. We detected the binding of IgG on the plate surface by using an anti-human IgG horseradish peroxidase antibody conjugate (Sigma-Aldrich, https://www.sigmaaldrich.com) and 3,3′,5,5′-etramethylbenzidine (Europa Bioproducts Ltd, https://www.europa-bioproducts.com). We analyzed samples in duplicate and evaluated optical density at 450 nm (OD_450_) data by dividing average OD_450_ values for individual samples by average OD_450_ of a known calibrator with negative antibody levels (T/N ratio). We defined results of 4 to <5 as equivocal and >5 as reactive. We defined samples as reactive for each assay independently.

#### Assay Validation

Because of the speed with which the assays have been developed, limited validation has been conducted by the manufacturers. We therefore used panels created by PHE and managed by the SEU to validate the assays (*7*,*8*)

#### Population Data

We weighted overall prevalence estimates for age. We based these estimates on population data from the Office for National Statistics (*9*).

### Statistical Analysis

We calculated observed prevalence (prev_obs_) by age group, sex, and time with 95% exact CIs. In these calculations, all results falling into the equivocal range of the assays were included as negative. Analyses were conducted in Stata 14 (StataCorp, https://www.stata.com).

We corrected observed prevalence to account for the sensitivity and specificity of the assays by using an adjusted prevalence (prev_adj_) related to the observed prevalence as follows:

Prev_obs_ = Se × prev_adj_ + (1 – Sp) × (1 – prev_adj_),

where Se denotes sensitivity, Sp denotes specificity, and prev_obs_ denotes the observed prevalence (*10*,*11*). We solved this relationship within a Bayesian model, along with the sampling distribution for reactive tests *n^+^*≈binomial(N, prev_obs_) and using a beta(0.5,0.5) prior for the adjusted prevalence prev_adj_. We included sensitivity and specificity, which were based on positivity in convalescent and baseline serum samples, in our Bayesian model each by way of a conjugate beta-binomial model with a beta(0.5,0.5) prior, thus accounting for uncertainty of their actual value. We generated uncertainty and credible intervals by using Markov Chain Monte Carlo simulations with 500,000 iterations after a burn-in of 1,000 iterations and a thinning interval of 5, using the NIMBLE package in R software (*12*,*13*).

## Results

**Figure 1:**
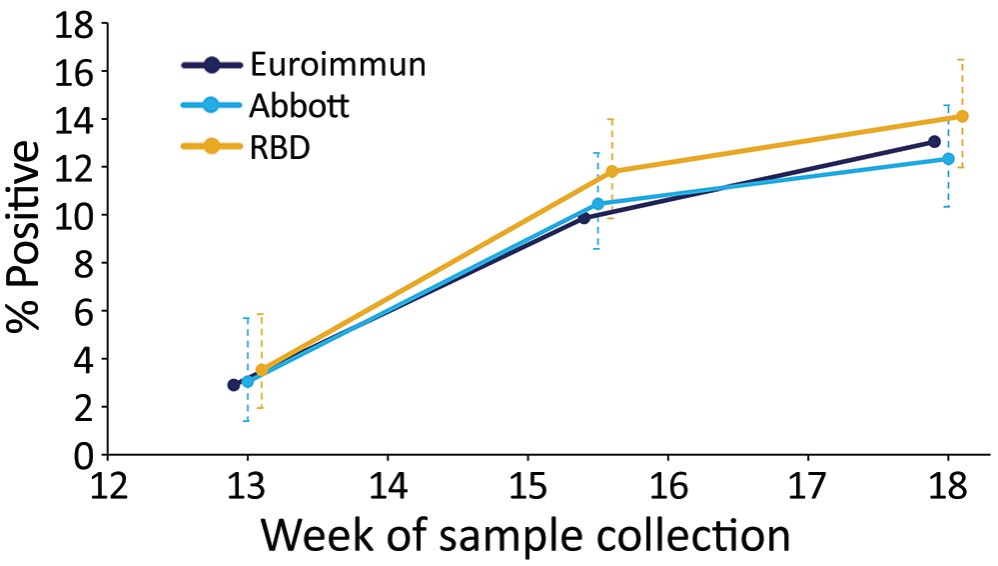
Percentage of reactive test results (unadjusted) for severe acute respiratory syndrome coronavirus 2 Ig in serum samples, by assay and epidemiologic week of sample collection (weeks 13, 15–16, and 18), London, UK, 2020. Error bars indicate 95% CIs. RBD, receptor-binding domain.

The overall test positivity based on the Euroimmun assay was 2.9% (95% CI 1.8%–4.4%) in week 13, 9.9% (95% CI 8.2%–11.8%) in weeks 15–16, and 13.0% (95% CI 11.0%–15.3%) in week 18. Consecutive differences between the proportion reactive at the 3 timepoints reduced over time, from 7.0% (95% CI 4.8%–9.1%) during week 13 to weeks 15–16 to 3.2% (95% CI 0.4%–5.9%) during weeks 15–16 to week 18 (Figure 1; Appendix Table 1).

In comparison, results from the RBD and Abbott assays had higher positivity at week 13, RBD at 3.5% (95% CI 1.9%–5.9%) and Abbot at 3.0% (95% CI 1.4%–5.6%), compared with a positivity of 2.9% with the Euroimmun assay (Figure 1; Appendix Table 1). The number of samples tested using each assay varied considerably for this first timepoint. At week 18 the RBD test had highest positivity at 14.1% (95% 12.0%–16.5%) and Abbott the lowest at 12.3% (95% CI 10.3%–14.6%) (Figure 1; Appendix Table 1), although the differences in positivity estimated by the 3 assays were not significantly different at each of the 3 periods, producing overlapping CIs. We tested a smaller number of donor samples from week 13 using the Abbott assay. The geographic spread of these samples was a little more concentrated in inner London compared with the overall sample collection (Appendix Table 2) but reasonably representative in terms of age (Appendix Table 3).

After adjustment for sensitivity and specificity, Euroimmun had the highest adjusted prevalence in week 18 at 14.9%, compared with 13.3% for the Abbot assay and 13.4% for the RBD assay (Appendix Table 1). In weeks 15–16, adjusted prevalence was similar among the 3 assays: 10.9% for RBD, 11.0% for Euroimmun, and 11.3% for the Abbott assay. In week 13, adjusted prevalence was lowest for RBD at 1.5% and highest for the Abbott at 3.1%.

**Figure 2:**
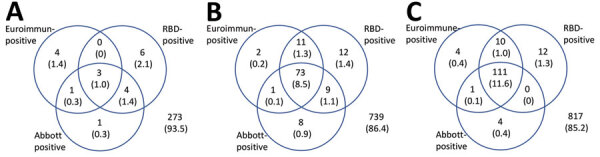
Results for serum samples tested for severe acute respiratory syndrome coronavirus 2 Ig with all 3 assays, by epidemiologic week of sample collection (weeks 13,15–16,and 18), London, UK, 2020. Values are no. (%) of reactive test results. RBD, receptor-binding domain.

Venn diagrams show the results for samples tested by all assays for the 3 timepoints (Figure 2). Unadjusted prevalence based on a highly specific endpoint requiring all assays to be reactive was 1.0%, 8.5% and 11.6% at the 3 timepoints, whereas if based on a highly sensitive endpoint of any assay reactive prevalence was 6.5%, 13.6%, and 14.8%. The RBD assay gave the most reactive results, but this tendency can be explained by its lower specificity (Appendix Table 5). If a criterion of reactive by Abbott or Euroimmun were used (to maintain good specificity and increase sensitivity), then unadjusted prevalence would be 4.5%, 12.2%, and 13.6% for the 3 timepoints. These values compare with an unadjusted prevalence of 2.9%, 9.9%, and 13.0% for the Euroimmun assay alone. To adjust prevalence based on assay combinations, the sensitivity and specificity of these combinations is also required, but the validation data did not have all assays tested on the same negative samples to enable this calculation.

**Figure 3:**
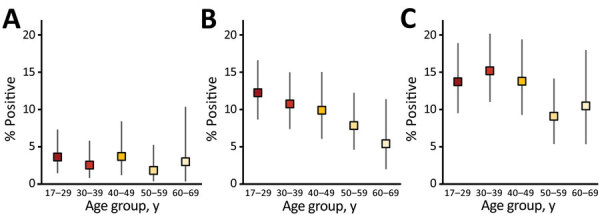
Percentage of reactive Euroimmun test results for severe acute respiratory syndrome coronavirus 2 Ig, by age group (unadjusted prevalence) and epidemiologic week of sample collection, London, United Kingdom, 2020. A) Week 13 (week beginning March 23); B) weeks 15–16; C) week 18.

The analysis shows an important age effect, a decreasing prevalence with increasing age group at weeks 15–16 (Figure 3; Appendix Table 3), based on the Euroimmun assay. Comparisons by age were not interpretable for the earlier timepoint (week 13) because of the low number of donor samples from persons in older age groups. At week 18, the difference in prevalence by age group was less pronounced, showing little difference between age groups <50 years and an increased prevalence in older age groups. When comparing age effects by assay, this effect was most pronounced in the RBD assay results and least pronounced in the Abbott assay results (Appendix Table 3).

Although prevalence estimates from all 3 assays indicate a slightly higher prevalence among men than women in week 13 (Appendix Table 6), a more pronounced gender effect appears to have occurred by weeks 15–16, when prevalence was higher in younger women than in men and older women. This difference was no longer observed by week 18, when prevalence was similar.

## Discussion

We demonstrate the value of using 3 serologic assays targeting different proteins for evaluating seroprevalence of SARS-CoV-2 and for understanding the evolution of the epidemic in London and the effects of physical distancing measures. Our results show that overall trends in prevalence estimates are similar across all 3 assays; however, we observed some notable differences. The sensitivity analysis indicates that the assay targeting the nucleoprotein identifies early infections; the assays targeting the spike protein are more reliable in picking up late infections. These results are similar to observations made by other groups (*14*). Including samples that were positive on the nucleoprotein-based assay with those reactive on a spike-based assay increased unadjusted prevalence by 1.6%, 2.3%, and 0.6% for the 3 periods.

Understanding the changes in sensitivity of serologic assays over time is also critical in interpreting seroprevalence data, particularly taking into account recent data that have indicated differential waning patterns for antibodies that have different targets (*14*). These findings also demonstrate the value in combining data from different serologic assays with different target proteins for determining seroprevalence.

We show that, in London, ≈14% donors had evidence of infection by week 18, the highest for any region of England. This pattern is consistent with data from other surveillance systems, including numbers of cases, hospitalizations, and deaths (*15*). These results also confirm transmission slowed substantially after lockdown measures were put in place, plateauing from weeks 15–16 to 18. Given the time required to develop an antibody response and the fact that donors are excluded from donating for a minimum of 14 days after an acute illness, these prevalence estimates reflect the situation >2–4 weeks before the collection date. Therefore, increases observed from weeks 13 to 15–16 reflect the situation before the effects of lockdown measures fully taking effect, and results from early May reflect incidence from early to mid-April.

Our analysis shows a very pronounced age difference among adult age groups, particularly for samples taken in weeks 15–16, which probably reflect the epidemic dynamics under normal social-mixing patterns in a high-transmission situation (*16*), given the fact that these results were too early to have been affected by lockdown. Those findings suggest that young adults in London were infected earlier in the epidemic and older age groups affected later. The mixing patterns during lockdown have substantially changed, including less frequent contact with persons in the same age groups (i.e., less age-assortative mixing); fewer daily contacts overall; and more intergenerational mixing among persons >30 years of age, probably reflecting household compositions in these age groups (*17*). These patterns might explain, in part, some of the observed differences in trends by age group.

For prevalence by sex, data from London suggest that young adult women had a higher risk for infection than men of the same age, particularly before lockdown measures were implemented. However, after lockdown, those differences became less pronounced. This finding might support the hypothesis that women of childbearing age were acquiring infection before men of a similar age group. Evidence to support the idea that children are key drivers of transmission is limited (*17*), and further work is needed to address potential explanations for such a difference, including higher intensity of exposure to children, higher frequencies of occupational caring roles for women compared with men, or both.

The availability of large volumes of donor samples on a weekly basis provided an attractive and valuable source of samples for seroprevalence estimates. However, adult donors are not representative of the general population and are likely to be less ethnically diverse, of higher socioeconomic status, and healthier than the wider population (*18*,*19*), all of which might lead to an underestimate in population prevalence. Although donors >70 years of age were excluded from donation, increased pathogenicity of SARS-CoV-2 with age might have resulted in an increased proportion of infected older donors being hospitalized and thus not available for blood donation. This tendency could result in an underestimate of seroprevalence in the oldest age groups.

Changes in the precise locations of sampling within regions at different periods have been observed, and this lack of consistent sampling needs to be considered when interpreting any changes over time. For example, because of limited volumes, a smaller number of donor samples from week 13 were tested using the Abbott assay. We demonstrate similar results for using 3 different assays independently and adjusting for the estimated sensitivity and specificity of the assays. We did not attempt to estimate adjusted prevalence on a combination of assays results or on the basis of changing assays cutoffs because more validation data on using multiple assays would be needed, and those data would probably indicate a pattern similar to that observed with the individual assays.

A range of interactions might have contributed to our results; further work is needed to understand the effect of age on antibody kinetics and the effect of age on different aspects of various assays, including sensitivity over time. These factors highlight the complexity inherent in interpreting seroprevalence surveys.

Despite those limitations, these results from testing blood donors have provided valuable intelligence regarding the progression of the epidemic among adults in London. Our results show that using multiple serologic assays targeting different proteins is probably informative as we try to determine the interplay between antibody kinetics and transmission dynamics in the population over time. Seroepidemiologic studies that rely on a single assay or have a single target risk incomplete ascertainment of the actual number of infections within the population.

Appendix Additional information about seroprevalence of SARS-CoV-2 among blood donors and changes after introduction of public health and social measures, London, England.
