# Supplementary material for: Seroprevalence of SARS-CoV-2 among Blood Donors and Changes after Introduction of Public Health and Social Measures, London, UK
Source: Emerg Infect Dis. 2021 Jul;27(7):1795–801. doi: 10.3201/eid2707.203167 (PMC8237903; doi:10.3201/eid2707.203167)
Supplement: Appendix — Additional information about seroprevalence of SARS-CoV-2 among blood donors and changes after introduction of public health and social measures, London, England. [file 20-3167-Techapp-s1.pdf]

Article DOI: https://doi.org/10.3201/eid2707.203167

Seroprevalence of SARS-CoV-2 among Blood Donors and Changes after Introduction of Public Health and Social Measures, London, England

Appendix

Assay Validation

Appendix Table 4 and 5 show the results from the panels for assessing sensitivity and specificity. For Euroimmun, sensitivity was 82.5% (95% CI 75.7–88.0), for Abbott 91.4% (95% CI 85.8%–95.4%) and for RBD 92.5% (95% CI 87.3%–96.1%). Within the hospitalized patients 100% were reactive by Abbott (23/23) and RBD (28/28) and 89% (25/28) by Euroimmun suggesting higher sensitivity in these cases. Specificity was very high for Euroimmun (99.1%) and Abbott (99.8%), and a little lower for RBD (98.0%). There were no significant differences in specificity between the panels for the same assay, this included RBD where there was a 1.1% lower specificity in the children's panel compared to the adult's panel (p = 0.32, Fisher exact test). This suggests that combining the adult's and children's data for prevalence adjustment on adults is reasonable.

Model Code Used to Adjust Prevalence for Sensitivity and Specificity

The following BUGS language model code was used to adjust for sensitivity and specificity, using the NIMBLE package in R.

Jeffreys Beta(0.5,0.5) prior on sensitivity (sens) and specificity (spec) gives the following posteriors

TP = true positives, FN = false negatives, TN = true negatives, FP = false positives

sens ~ dbeta(TP+0.5, FN+0.5)

```
spec ~ dbeta(TN+0.5, FP+0.5)

# pos = number of positive tests, N = total samples tested, obs_prev = observed prevalence

pos ~ dbin(obs_prev, N)

# adjustment, true_prev = adjusted prevalence

obs_prev <- true_prev * sens + (1-true_prev)*(1-spec)

# prior on adjusted prevalence

true_prev ~ dbeta(0.5, 0.5)
```

Appendix Table 1. Observed and adjusted prevalence by week of sample, in London, using the Abbott, Euroimmun and RBD assays

Week	Date range	Assay	Neg	Equiv	Pos	Total	Pos, % (95 CI)	Adjusted prevalence, % (95 CrI)
13	26–27 Mar	Abbott	289	1	9	299	3.0 (1.4–5.6)	3.1 (1.3–5.7)
		EuroImmun	724	11	22	757	2.9 (1.8–4.4)	2.3 (0.4–4.3)
		RBD	373	9	14	396	3.5 (1.9–5.9)	1.5 (0.0–4.0)
15–16	9–13 Apr	Abbott	834	15	99	948	10.4 (8.6–12.6)	11.3 (9.2–13.6)
		EuroImmun	963	15	107	1085	9.9 (8.2–11.8)	11.0 (8.6–13.6)
		RBD	848	19	116	983	11.8 (9.8–14.0)	10.9 (8.5–13.4)
18	1–3 May	Abbott	836	17	120	973	12.3 (10.3–14.6)	13.3 (11.1–15.8)
		EuroImmun	837	10	127	974	13.0 (11.0–15.3)	14.9 (12.1–18.0)
		RBD	807	21	136	964	14.1 (12.0–16.5)	13.4 (10.9–16.2)

Appendix Table 2. Count (%) of samples analyzed by assay and London region of residence for each period of sampling

		Inner London		Outer London	
Week	Assay	North	South	North	South
13	Abbott	61 (20)	118 (39)	82 (27)	38 (13)
	EuroImmun	191 (26)	217 (29)	209 (28)	140 (18)
	RBD	85 (22)	138 (35)	128 (32)	45 (11)
15–16	Abbott	247 (26)	269 (28)	185 (20)	247 (26)
	EuroImmun	275 (26)	329 (30)	209 (19)	272 (25)
	RBD	244 (25)	318 (32)	151 (15)	270 (27)
18	Abbott	338 (35)	316 (32)	157 (16)	162 (17)
	EuroImmun	337 (35)	318 (33)	158 (16)	161 (17)
	RBD	336 (35)	313 (32)	154 (16)	161 (17)

Appendix Table 3. Observed prevalence by age, and week of sample, in London, using the Abbott, Euroimmun and RBD assays*

			Abbott		EuroImmun		RBD	
Week	Date range	Age group	Pos/total	Pos, % (95 CI)	Pos/Total	Pos, % (95 CI)	Pos/Total	Pos, % (95 CI)
13	26–27 Mar	17–29	3/85	3.5 (0.7–10.0)	7/193	3.6 (1.5–7.3)	7/110	6.4 (2.6–12.7)
		30–39	2/79	2.5 (0.3–8.8)	5/197	2.5 (0.8–5.8)	3/103	2.9 (0.6–8.3)
		40–49	2/44	4.5 (0.6–15.5)	5/135	3.7 (1.2–8.4)	2/65	3.1 (0.4–10.7)
		50–59	1/62	1.6 (0.0–8.7)	3/164	1.8 (0.4–5.3)	1/79	1.3 (0.0–6.9)
		60–69	1/28	3.6 (0.1–18.3)	2/67	3.0 (0.4–10.4)	1/38	2.6 (0.1–13.8)
15–16	9–13 Apr	17–29	30/240	12.5 (8.6–17.4)	35/286	12.2 (8.7–16.6)	41/264	15.5 (11.4–20.5)
		30–39	31/248	12.5 (8.7–17.3)	30/279	10.8 (7.4–15.0)	37/255	14.5 (10.4–19.4)
		40–49	19/170	11.2 (6.9–16.9)	19/192	9.9 (6.1–15.0)	17/167	10.2 (6.0–15.8)
		50–59	14/193	7.3 (4.0–11.9)	17/217	7.8 (4.6–12.2)	16/196	8.2 (4.7–12.9)
		60–69	5/97	5.2 (1.7–11.6)	6/111	5.4 (2.0–11.4)	5/101	5.0 (1.6–11.2)
18	1–3 May	17–29	25/225	11.1 (7.3–16.0)	31/226	13.7 (9.5–18.9)	33/223	14.8 (10.4–20.1)
		30–39	40/259	15.4 (11.3–20.4)	39/257	15.2 (11.0–20.2)	44/255	17.3 (12.8–22.5)
		40–49	25/196	12.8 (8.4–18.3)	27/196	13.8 (9.3–19.4)	28/194	14.4 (9.8–20.2)
		50–59	17/185	9.2 (5.4–14.3)	17/187	9.1 (5.4–14.2)	18/184	9.8 (5.9–15.0)
		60–69	11/105	10.5 (5.3–18.0)	11/105	10.5 (5.3–18.0)	11/105	10.5 (5.3–18.0)

*One person in week 13 and 3 persons in week 18 were aged 70+ and have not been counted in this analysis.

Appendix Table 4. Sensitivity of Euroimmun, Abbott and RBD assays by weeks since onset or PCR confirmation. Equivocal results are treated at negative for sensitivity calculation.

Assay	Dataset	Reactive	Equivocal	Negative	Total	Sensitivity, % (95 CI)
Euroimmun	Conv (21–27 d)	23	5	4	32	71.9 (53.3–86.3)
	Conv (28–34 d)	65	2	7	74	87.8 (78.2–94.3)
	Conv (35–41 d)	33	2	5	40	82.5 (67.2–92.7)
	Conv (≥42 d)	11	0	3	14	78.6 (49.2–95.3)
	Total	132	9	19	160	82.5 (75.7–88.0)
Abbott	Conv (21–27 d)	27	0	0	27	100 (87.2–100)
	Conv (28–34 d)	63	3	4	70	90 (80.5–95.9)
	Conv (35–41 d)	38	1	2	41	92.7 (80.1–98.5)
	Conv (≥42 d)	11	2	1	14	78.6 (49.2–95.3)
	Total	139	6	7	152	91.4 (85.8–95.4)
RBD	Conv (21–27 d)	30	0	2	32	93.8 (79.2–99.2)
	Conv (28–34 d)	69	2	3	74	93.2 (84.9–97.8)
	Conv (35–41 d)	38	2	1	41	92.7 (80.1–98.5)
	Conv (≥42 d)	12	0	2	14	85.7 (57.2–98.2)
	Total	149	4	8	161	92.5 (87.3–96.1)

Appendix Table 5. Specificity of Euroimmun, Abbott and RBD assays in 2018 panels from SEU and RCGP-RSC. Equivocal results are treated as negative for specificity calculation.

Assay	Panel	Median age (range)	Reactive	Equivocal	Negative	Total	Specificity, % (95 CI)
Euroimmun	RCGP RSC adult panel	52 (18–93)	1	0	174	175	99.4 (96.9–100)
	SEU adult panel	42 (16–83)	4	6	389	399	99 (97.5–99.7)
	Total	44 (16–93)	5	6	563	574	99.1 (98–99.7)
Abbott	SEU age 1–30 panel	17 (1–30)	2	6	743	751	99.7 (99–100)
	SEU adult panel	42 (16–84)	0	2	393	395	100 (99.1–100)
	Total	22 (1–84)	2	8	1,136	1,146	99.8 (99.4–100)
RBD	RCGP RSC adult panel	54 (18–93)	3	3	258	264	98.9 (96.7–99.8)
	SEU age 1–30 panel	16 (1–30)	19	12	827	858	97.8 (96.6–98.7)
	Total	20 (1–93)	22	15	1,085	1,122	98 (97–98.8)

Appendix Table 6. Observed prevalence by gender and broad age group (17–44, 45–69), and week of sample, in London, using the Abbott, Euroimmun and RBD assays

				Abbott		EuroImmun		RBD	
Week	Date range	Age	Sex	Pos/ Total	Pos, % (95 CI)	Pos/ Total	Pos, % (95 CI)	Pos/ Total	Pos, % (95 CI)
13	26–27 Mar	17–44	F	3/96	3.1 (0.6–8.9)	6/225	2.7 (1.0–5.7)	5/126	4.0 (1.3–9.0)
		17–44	M	3/88	3.4 (0.7–9.6)	8/222	3.6 (1.6–7.0)	7/119	5.9 (2.4–11.7)
		45–69	F	0/48	0.0 (0.0–7.4)	1/132	0.8 (0.0–4.1)	0/64	0.0 (0.0–5.6)
		45–69	M	3/66	4.5 (0.9–12.7)	7/177	4.0 (1.6–8.0)	2/86	2.3 (0.3–8.1)
15–16	9–13 Apr	17–44	F	47/302	15.6 (11.7–20.2)	48/334	14.4 (10.8–18.6)	53/304	17.4 (13.3–22.2)
		17–44	M	24/277	8.7 (5.6–12.6)	27/334	8.1 (5.4–11.5)	33/306	10.8 (7.5–14.8)
		45–69	F	9/147	6.1 (2.8–11.3)	9/155	5.8 (2.7–10.7)	10/134	7.5 (3.6–13.3)
		45–69	M	19/222	8.6 (5.2–13.0)	23/262	8.8 (5.6–12.9)	20/239	8.4 (5.2–12.6)
18	1–3 May	17–44	F	49/337	14.5 (11.0–18.8)	47/336	14.0 (10.5–18.2)	54/333	16.2 (12.4–20.6)
		17–44	M	32/251	12.7(8.9–17.5)	39/251	15.5 (11.3–20.6)	39/248	15.7 (11.4–20.9)
		45–69	F	15/188	8.0 (4.5–12.8)	16/188	8.5 (4.9–13.5)	17/187	9.1 (5.4–14.2)
		45–69	M	22/194	11.3 (7.2–16.7)	23/196	11.7 (7.6–17.1)	24/193	12.4 (8.1–17.9)
